# P-1087. Novel Antisense Wide-Range Peptide Nucleic Acids Coated on Polystyrene Plates to Prevent Bacterial Biofilm

**DOI:** 10.1093/ofid/ofae631.1275

**Published:** 2025-01-29

**Authors:** Hannah Karp, Elizabeth Nowak, Gillian Su, Nihan Akguc, Michael Schulz, Nammalwar Sriranganathan, Jayasimha Rao

**Affiliations:** Virginia Tech Carilion School of Medicine, Roanoke, Virginia; Carilion Clinic, Roanoke, Virginia; Virginia Tech, Blacksburg, Virginia; Department of Biomedical Sciences and Pathology VMC of VM, Blacksburg, Virginia; Virginia Tech, Blacksburg, Virginia; Virginia Polytechnic Institute and State University, Blacksburg, Virginia; Carilion Clinic/Virginia Tech Carilion School of Medicine, Roanoke, Virginia

## Abstract

**Background:**

Biofilms are three dimensional communities of microorganisms that are encased in self-produced extracellular polymeric substances. Biofilms play a key role in device-associated infections, including catheter-associated urinary tract infections, largely because they protect microorganisms from standard antimicrobial therapies. Antisense synthetic peptide nucleic acids (PNAs) are an emerging approach to inhibiting specific components of gene expression. Several regulatory and motility genes are relatively conserved across biofilm forming Gram-negative bacilli, such as *rpoS*, *amrZ*, *rsmA*, and *motA*. In the present study, wide range PNAs (wrPNAs) were developed by our group to exploit these conserved targets, offering a mechanism for precise, yet broad, biofilm inhibition. The impact of wrPNAs on Gram-negative biofilms and bacterial viability were investigated.

**Figure:**
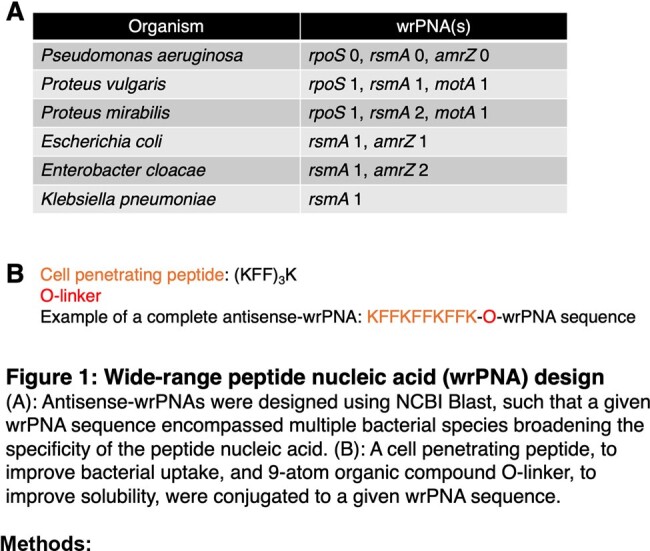

**Methods:**

**wrPNAs**: Antisense-wrPNAs (patent pending) were designed using NCBI Blast to select a region inclusive of the start codon. The wrPNAs ranged from 12 to 14 basepairs, conjugated with a cell-penetrating peptide and O-linker, and were synthesized by PNA Bio (Figure 1).

**Quantify biofilm biomass**: Bacterial isolates were grown in a 96-well polystyrene plate in minimal salt medium with wrPNA treatment (10 µM each) and incubated at 30˚C for 24hr in a humid chamber. Biofilms were stained with 0.1% crystal violet, solubilized in 33% glacial acetic acid, and measured at OD_590 nm_.

**Quantify bacterial viability**: 10-fold serial dilutions and direct stamping onto agar plates.

**Statistical analysis**: P values were determined using unpaired t-tests or one-way ANOVA with followup tests comparing the mean of each column to the control column (alpha = 0.05).

**Figure:**
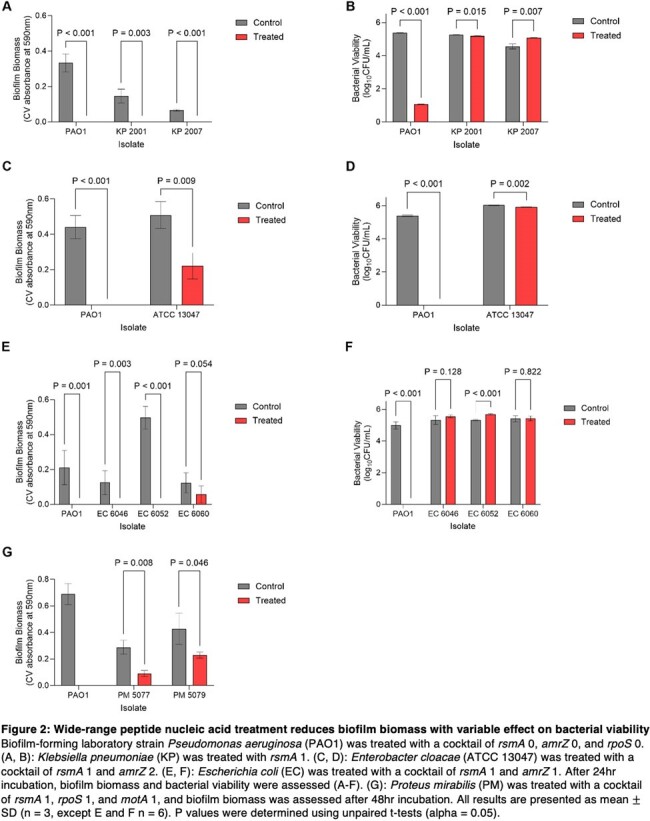

**Results:**

wrPNA treatment significantly reduced biofilm biomass for their intended species (Figure 2). A cocktail of PNAs had a bactericidal effect on *Pseudomonas aeruginosa*, but wrPNA treatment had minimal effect on bacterial viability for the other Gram-negative bacilli tested (Figure 2).

**Conclusion:**

wrPNAs against global regulator genes and a motility regular gene reduced biofilm biomass for their intended species. Future work will look at combination therapy to improve bactericidal effects as well as methods for coating treatments onto catheter and device surfaces.

**Disclosures:**

**All Authors**: No reported disclosures

